# With Our Editors

**Published:** 1907-07-15

**Authors:** 


### WITH OUR EDITORS.

**FADS.** The programs of the various State Societies indicate that we are following after fads. The enthusiast in any branch of dentistry is as necessary as the conservative, and has been an important factor in the developement of the highest ideals. It is his mission to draw you out of the rut that has been your comfortable resting place so long that you had forgotten when and how you got in. This is the real value of our State Associations, the bringing men of different methods and ideas together to compare, weigh, and assimilate. The inlay man has the center of the floor just now, but we are beginning to find their limitations. The first demonstration of gold inlay before the Kansas Association was given either in 1885 or '86, and was generally condemned on the ground of the weakness of a cement joint. It was a new factor in the methods of saving teeth and one which the profession was slow in adopting. It and the porcelain inlay have had to fight for proper recognition and one of their great handicaps has been the extravagant claims of their friends. This is best illustrated by relating an incident that happened at one of the I. S. D. F. banquets. None of the speakers seemed to please one of the members, and he kept whispering to his neighbor "Wait until Dr...............'s turn comes and you will hear a *speech*." This enthusiast certainly received a shock when his friend Dr............arose and proceeded to read a very carefully prepared typewritten extemporaneous after dinner speech. The speaker failed to "make good" the reputation given him by his friend. Not that he did not have the ability to make a fine after dinner speech, but knowing he was to speak to an audience who were strangers to him and that he would suffer from stage fright, he decided not to take any chance of forgetting his lines and the absolutely safe thing to do would be to read his speech, which he did. Other special methods have suffered in like manner from their friends, and it is sometimes difficult to be "Mejum,"—and not be "Too Mejum." While we have been trying this, that or some other material or method the burden-bearers (amalgam and gold), have continued to make good in a large percentage of the operations for saving teeth. The charge has been made that, as a profession, we were inclined to use such material as we could receive the greatest fee for its use.

There is no doubt that there is no class of professional men who strive harder to return to their patients value received than the members of the dental profession. One of the important items brought out in discussion at the Kansas meeting was that we must readjust our ideas of charges, if we have not already done so. That the material used should not be the basis of our charges, but our patients should be educated to understand they were paying for professional services. This point of view would do much to clear some of the problems in the minds of dentists themselves and establish a reasonable foundation for a correct basis of fees.

The jeweler, silversmith, goldsmith and dental dealer can *sell* materials, but without the skill of the dentist they are of no use to the patient in the matter of saving teeth. When we get this thoroughly in our minds as a profession and *live up* to the right conception of our relation to the patient, it will have done much to put the bargain-giver and and the bargain-hunter into a class distinctly by themselves and into which no self-respecting dentist will be found. While fees are under discussion, just a word about the *commission* business. The Kansas Association passed a resolution against the giving or taklng commissions. If one really wished to go into the commission business, it would have been a great deal better to have dealt in cattle, sheep, hogs, produce or some line that there was something doing in all the time, instead of *piloting* some patient into the hands of the specialist who would give *you* the best commission. There is a vast difference between the live stock commission merchant and the dentist. The one sends the pig to market and it comes back to you in a few weeks in the form of fancy cured bacon and the other sends his patient to a specialist upon whose *skill* (not the commission) depends their future health and happiness.—*F. Q. Hetrick, in Western Journal.*

**CASE VS. DEWEY, ANGLE, ET AL.** *The Dental Cosmos* for May, 1907, contains a very spirited article by Dr. Calvin S. Case of Chicago, on the subject of "Rise and Developement of the Intermaxillary'Force." Dr. Case maintains that he is the originator of the use of an elastic band from the upper to the lower teeth, or *vice versa*,, by which teeth of one jaw are made the anchorage for movement of the opposite set, and which he calls intermaxillary anchorage or intermaxillary force.

This matter has been up for discussion before, and Dr. Case, Dr. Angle, Dr. Baker and Dr. Dewey have all figured therein.

Dr. Angle, in his book, gives credit to Dr. Baker for the use of an elastic band in the same manner as described, and gives it the name of Baker anchorage. Dr. Dewey has an article on this subject in *The Cosmos* for May, 1906. The present article by Dr. Case is a combined reply, criticism, and defiance to Drs. Dewey, Angle, Baker and the "new school" of orthodontia in general.

Dr. Case is well known in dental literature as a "scrapper," and he certainly does justice to his reputation in this instance, for he lands rights, lefts, uppercuts, and some very vicious swings all over "the other fellow." He also sidesteps nicely at certain places, and possibly ducks the real issue a little on certain other points. Dr. Dewey gets jabs, punches, hunches and taffy, all in the same article.

We need not be concerned over the welfare of any of the principals in this affair, as they are all abundantly able to take care of themselves. Neither are we concerned over the exact minute when one first made use of this, or the other made use of that. If the truth was only known, it is likely the same thing was used by someone else long before either of the present claimants knew the alphabet. And we further need not care the slightest whether this thing is dubbed Baker anchorage, intermaxillary force, or what not. We ourselves will call it by whatever name we shall decide best describes it—and there's the end of that.

While the "scrap" over this anchorage may interest outsiders enough that they may look on and smile at the "frantic efforts" of the participants, yet no one outside of the immediate principals cares three whoops for the whole thing, one way or the other. What would be of interest and of real use would be full descriptions and illustrations of the use of this device, so that every possible benefit to humanity might come from it. But no! We have instead the spectacle of men thinking more of their own selfish interests, with one claiming "It's mine; I found it first," while the other yells: "'Taint, either; you know its mine."

Dentists—and especially orthodontists—sometimes get all puffed up and claim they are real scientists. Does this sort of behavior over a little petty thing (for it is a little, miserable, petty mechanical detail) tend to prove it. As a mere matter of contrast let the words and conduct of Sir Isaac Newton be recalled when he told of the discovery of the law of gravitation, or Charles Darwin when he first announced his conclusions regarding the origin of species, both of which were set forth with a modesty of statement that our "scientists" might well pattern after, and which, too, were possibly fully equal in importance to the "inter-maxillary force" or "Baker anchorage."

WILLIAM J. BRADY, in *Western Journal.*

**A DEVICE FOR CASTING METALS UNDER PRESSURE; DESIGNED ESPECIALLY FOR GOLD INLAYS WITHOUT USING A MATRIX.**

In publishing this paper the Editor of this Department feels that he is presenting to the readers of the DENTIST'S MAGAZINE a simple and inexpensive apparatus for the construction of the ideal gold inlay, we will go a step farther and say the ideal gold filling.

After seeing Dr. Kenyon cast an inlay the writer and several others insisted that the doctor have an apparatus made for each of them. This was done, and within two hours after receiving and connecting up his machine, the writer had cast two perfectly fitting inlays for a practical case.

This statement is made to show the simplicity and accuracy of the machine herein described.

The apparatus consists of a base carrying a supporting column about ten inches high with two arms which support an upright plunger, a flask with a cover that closes practically air-tight, and an investment shaper. The plunger consists of two brass tubes which telescope. A small tube enters the casing of the plunger and the inner casing carries the cover for the flask at the lower end of the plunger. The telescoping tubes form a valve which is automatically opened by pressing the plunger down to close the flask. The shaper is a form on which the flask ring is inverted while the investment is being poured. It shapes the surface of the investment in the form of a funnel leading to the gate to the mold. This funnel is the crucible in which the metal is fused for the cast.

The process of making an inlay of gold with this outfit is described as follows:

Prepare the cavity in such form as you would for a gold inlay to be made by any other method.

The pattern or model.

Mold into the cavity, in exact form to represent the finished filling, a piece of specially prepared wax. It must contain nothing that will not wholly disappear when burned. Uncolored baseplate wax may be used if none better is at hand. It should be softened by dry heat and the cavity should be wet to prevent the wax from sticking. Finish the wax filling to a smooth surface and leave a slight overlap at the margins. Harden it with a few drops of cold water or a cold air blast and carefully remove it from the cavity. You have then a perfect pattern of the inlay you wish to construct. No gold or platinum matrix is necessary or of use in the process. To some convenient surface of this wax pattern attach the end of a little short wire—the sprue former—by slightly heating it, sticking it into the wax and quickly cooling. Insert the other end of the wire into the hole in the apex of the cone on the investment shaper.

The investing material must be verv fine and its fusing point must be very high. Peck's and Pelton's investment compounds answer the purpose fairly well, but would be better if ground finer. First a small amount should be mixed carefully to exclude bubbles and applied to the wax pattern with a camel's hair brush until it is about one-fourth of an inch thick around the wax and covering the wire and the point of the cone in which the wire rests. Care must be exercised to allow no air bubbles to lodge next to the wax. When this has slightly set the flask ring is placed on the shaper in such manner that the invested pattern is near its center and the space is filled in with another mix of the same investing compound, or any other that will not shrink or crack when heated to a high temperature. When the investment has hardened remove the shaper by a rotary motion, warm the sprue-wire and extract it also by a rotary motion.

Running the cast.

Heat the flask gently at first until the investment is dry and then as rapidly as desired till it is nearly or quite to red heat. The wax will have disappeared, leaving a perfect mold in which to run the cast.

While the mold is drying connect a rubber tubing from the compressed air supply to the intake on the side of the upright plunger and withdraw the inner tube, bareing the flask cover, about an inch. This closes the valve and the compressed air should now be turned on. Slide the plunger up till the intake tube is near the upper arm of the supporting column. Also connect a cylinder of oxygen or nitrous oxid gas to the air tube of any ordinary gas blow-pipe. A small needle-point flame about an inch or an inch and a half long is better than a larger one.

Next set the hot flask in position in the counter-sink under the plunger and place the gold in the funnel shaped depression over the mouth of the gate into the mold. Heat the gold until it is not only fused but boiling, or nearly so, then gently but quickly slide the plunger down till the cover closes firmly on the flask and the tubes are completely telescoped. This opens the valve allowing the compressed air to enter the flask, which instantly drives the melted gold down into the mold with such force that the finest lines and thinest edges are exactly reproduced in the casting as they appeared in the pattern. After about one minute the flask may be dropped into water to cool. The cast should now be taken from the mold and pickled by boiling in hydrochloric acid and cleaned with a stiff brush to remove all the investment, after which it is ready to be finished and polished.

One of the principal advantages of this method of making inlays is the ease and certainty with which contour and occlusion are correctly obtained. Also a large complicated case is as quickly handled as smaller cases, and the trouble and uncertainty of forming a matrix are dispensed with.

This apparatus may also be used to cast small partial plates, small pieces of bridge-work, bridge "dummies," and retaining appliances for orthodontia and pyorrhea cases.

In fact any appliance small enough to be contained in the flask may be cast in any of the metals or alloys ranging all the way from Mellotte's metal to platinum.

The ideas involved in this process are not of recent origin, but have recently been recalled to the attention of the profession by the successful work of Dr. Taggart, of Chicago, to whom great credit is due. The writer claims originality only for design of the apparatus herewith described and its special adaptation to this method of constructing fillings, bridges, etc.

*Dentists Magazine.*

